# Retraction: IGF-1 induces the epithelial-mesenchymal transition via Stat5 in hepatocellular carcinoma

**DOI:** 10.18632/oncotarget.28573

**Published:** 2024-08-05

**Authors:** Chuanzong Zhao, Qian Wang, Ben Wang, Qi Sun, Zhaobin He, Jianguo Hong, Florian Kuehn, Enyu Liu, Zongli Zhang

**Affiliations:** ^1^Department of General Surgery, Qilu Hospital of Shandong University, Jinan, P.R. China; ^2^Department of Cardiology, Shandong Provincial Hospital Affiliated to Shandong University, Jinan, P.R. China; ^3^Department of General, Thoracic, Vascular and Transplantation Surgery, University of Rostock, Rostock, Germany

**This article has been retracted:** In this paper, the Stat5 blots in Figure 2A contain an internal duplication. In addition, several western blot panels in Figure 7B are duplicates of those found in Figure 4B and 4C but representing different proteins, conditions and cell types, in an earlier published paper [1]. All the panels in Figure 4A and 4C, representing transwell assay data, are duplicates of those found in Figure 1 of an earlier published paper and describing completely different conditions and cell types [2]. Therefore, the Editorial decision has been made to retract this paper. All authors agreed with the decision.

Original article: Oncotarget. 2017; 8:111922–111930. 111922-111930. https://doi.org/10.18632/oncotarget.22952
